# Supplementary material for: Causal Relationship between Aging and Anorexia Nervosa: A White-Matter-Microstructure-Mediated Mendelian Randomization Analysis
Source: Biomedicines. 2024 Aug 16;12(8):1874. doi: 10.3390/biomedicines12081874 (PMC11351342; doi:10.3390/biomedicines12081874)
Supplement: Supplementary file 1 [file biomedicines-12-01874-s001.zip › Supplementary Figure/Figure S4/Singe SNP plot of MR analysis for TL to ubm-b-1809.pdf]

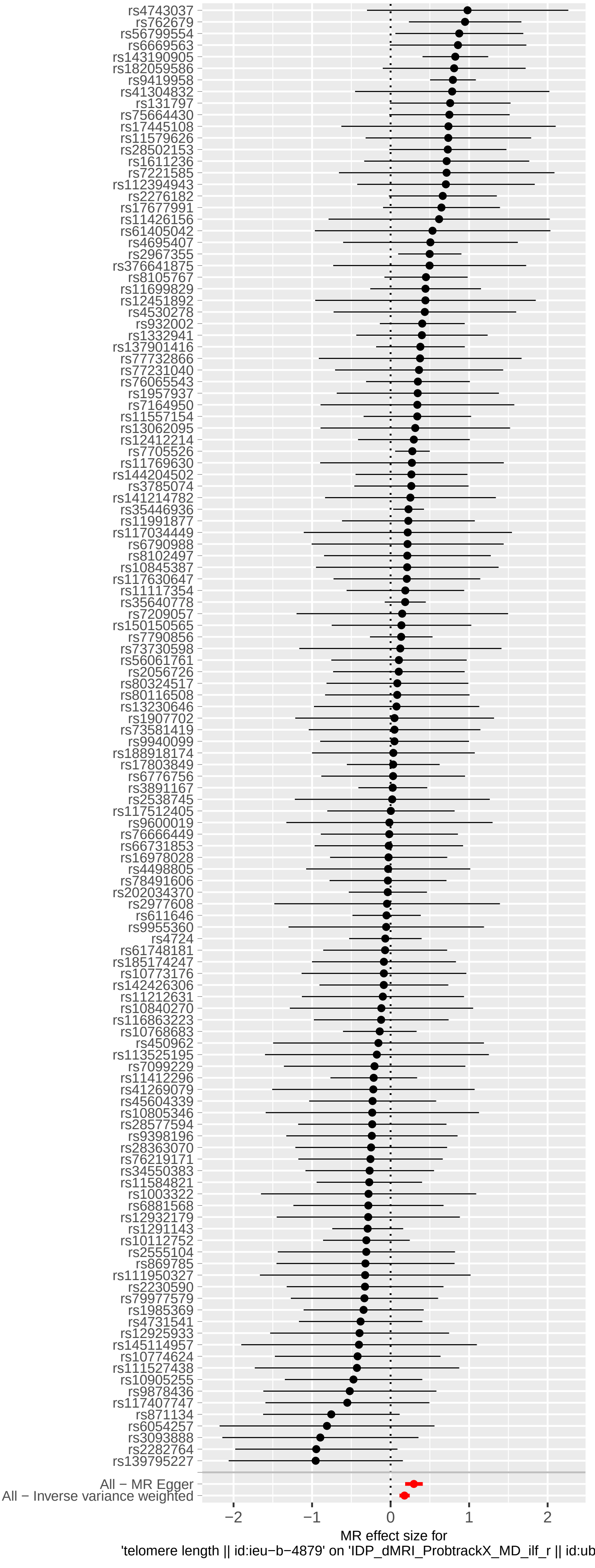
rs4743037
rs762679
rs56799554
rs6669563
rs143190905
rs182059586
rs9419958
rs41304832
rs131797
rs75664430
rs17445108
rs11579626
rs28502153
rs1611236
rs7221585
rs112394943
rs2276182
rs17677991
rs11426156
rs61405042
rs4695407
rs2967355
rs376641875
rs8105767
rs11699829
rs12451892
rs4530278
rs932002
rs1332941
rs137901416
rs77732866
rs77231040
rs76065543
rs1957937
rs7164950
rs11557154
rs13062095
rs12412214
rs7705526
rs11769630
rs144204502
rs3785074
rs141214782
rs35446936
rs11991877
rs117034449
rs6790988
rs8102497
rs10845387
rs117630647
rs11117354
rs35640778
rs7209057
rs150150565
rs7790856
rs73730598
rs56061761
rs2056726
rs80324517
rs80116508
rs13230646
rs1907702
rs73581419
rs9940099
rs188918174
rs17803849
rs6776756
rs3891167
rs2538745
rs117512405
rs9600019
rs76666449
rs66731853
rs16978028
rs4498805
rs78491606
rs202034370
rs2977608
rs611646
rs9955360
rs4724
rs61748181
rs185174247
rs10773176
rs142426306
rs11212631
rs10840270
rs116863223
rs10768683
rs450962
rs113525195
rs7099229
rs11412296
rs41269079
rs45604339
rs10805346
rs28577594
rs9398196
rs28363070
rs76219171
rs34550383
rs11584821
rs1003322
rs6881568
rs12932179
rs1291143
rs10112752
rs2555104
rs869785
rs111950327
rs2230590
rs79977579
rs1985369
rs4731541
rs12925933
rs145114957
rs10774624
rs111527438
rs10905255
rs9878436
rs117407747
rs871134
rs6054257
rs3093888
rs2282764
rs139795227
All − MR Egger
All − Inverse variance weighted
−2
−1
0
1
2
MR effect size for
'telomere length || id:ieu−b−4879' on 'IDP_dMRI_ProbtrackX_MD_ilf_r || id:ub
